# Retraction: Designing a novel visible-light-driven heterostructure Ni–ZnO/S-g-C_3_N_4_ photocatalyst for coloured pollutant degradation

**DOI:** 10.1039/d6ra90073a

**Published:** 2026-07-23

**Authors:** Ali Bahadur, Shahid Iqbal, Hashem O. Alsaab, Nasser S. Awwad, Hala A. Ibrahium

**Affiliations:** a Department of Transdisciplinary Studies, Graduate School of Convergence Science and Technology, Seoul National University Seoul 08826 South Korea; b Department of Chemistry, School of Natural Sciences (SNS), National University of Science and Technology (NUST) H-12 Islamabad 46000 Pakistan shahidiqbal.chem@sns.nust.edu.pk; c Department of Pharmaceutics and Pharmaceutical Technology, Taif University P. O. Box 11099 Taif 21944 Saudi Arabia; d Research Center for Advanced Materials Science (RCAMS), King Khalid University P. O. Box 9004 Abha, 61413 Saudi Arabia; e Department of Semi Pilot Plant, Nuclear Materials Authority P. O. Box 530 El Maadi Egypt

## Abstract

Retraction of ‘Designing a novel visible-light-driven heterostructure Ni–ZnO/S-g-C_3_N_4_ photocatalyst for coloured pollutant degradation’ by Ali Bahadur *et al., RSC Adv.*, 2021, **11**, 36518–36527, https://doi.org/10.1039/D0RA09390D.

The Royal Society of Chemistry hereby wholly retracts this *RSC Advances* article due to concerns with the reliability of the data.

The FTIR spectra in Fig. 5 were first published in ref. 1 and described as a different experiment.

The XRD patterns for 0NZO-30-SCN and 4NZO in Fig. 2 are partially identical.

The XRD patterns for 4NZO-30-SCN before reaction and 4NZO-30-SCN after reaction in Fig. 8b are partially identical.

The authors provided a response and new data but they did not address the concerns and they were not able to provide the original raw data.

Given the significance of these concerns, the findings presented in this paper are no longer reliable.

Shahid Iqbal opposes this retraction decision. The other authors did not respond to any correspondence regarding the retraction of this article.

This retraction supersedes the information provided in the Expression of Concern related to this article.

Signed: Laura Fisher, Executive Editor, *RSC Advances*

Date: 16th July 2026

**References**

1. M. A. Qamar, S. Shahid, M. Javed, S. Iqbal, M. Sher, A. Bahadur, M. M. AL-Anazy, A. Laref and D. Li, *Colloids Surf., A*, 2021, **614**, 126176, 10.1016/j.colsurfa.2021.126176

